# Linking Species Functional Traits to Specific Biogeochemical Processes under Trawling Pressure

**DOI:** 10.3390/biology11101378

**Published:** 2022-09-21

**Authors:** Irini Tsikopoulou, Christopher J. Smith, Konstantia Nadia Papadopoulou, Melanie C. Austen

**Affiliations:** 1 Hellenic Centre for Marine Research, P.O. Box 2214, 71003 Heraklion, Greece; 2 School of Biological and Marine Sciences, University of Plymouth, Plymouth PL4 8AA, UK

**Keywords:** oxygen flux, functional traits, trawling impact, seasonal fishery

## Abstract

**Simple Summary:**

Bottom trawls when fishing move over large areas with different parts of the gears physically impacting the sea bottom, including the trawling wires, doors, ground rope and net. In this way, the trawl nets remove animals from bottom waters, the sediment surface and shallow sub-surface. The animals that live in the sea bottom with their activities and lifestyle play an important role in major ecosystem processes such as nutrient cycling. In this study, we investigated the relationship between species functional characteristics and ecosystem functions under trawling pressure. Our results indicated that under trawling, more opportunistic lifestyles and deposit feeders were associated with the ecosystem processes while in the undisturbed areas these processes were connected with bioturbating and burrowing species. Finding these links helps scientists and policy makers to better predict the impact of fishing disturbance on marine environment and set appropriate thresholds for marine ecosystem impacts.

**Abstract:**

The impact of otter trawling on the relationship between functional traits of benthic invertebrates and specific biogeochemical processes were investigated in the oligotrophic Cretan Sea. The fishery is managed through a seasonal closure during the summer. During two seasons (winter and summer) replicate samples were taken from the field from a commercial trawl ground and an adjacent control area. Environmental parameters related to sediment biogeochemistry were measured including particulate organic carbon, sedimentary organic carbon, bottom water and sedimentary chlorophyll a and phaeopigment concentrations as well as benthic oxygen consumption. A significant impact of trawling was recorded only for bottom water chlorophyll and sedimentary organic carbon. Furthermore, the links between species traits and specific ecosystem processes were affected by trawling, highlighting the importance of unique functional modalities on ecosystem functioning. The traits that mostly influenced benthic biogeochemistry in the control sites were related to bioturbation and burrowing activities. In contrast, in the trawled sites, the associated traits were related to more opportunistic lifestyles and deposit feeding species that do not act as bioturbators. Thus, under trawling disturbance, this shift can decouple the species-sediment relations and affect nutrient cycling.

## 1. Introduction

Grouping benthic invertebrate species according to their functional identity is a widespread approach that has led to an improved understanding of the role of these species within a community [1]. It also provides a useful tool for linking the ecosystem processes with specific functions as biological traits analysis can provide a direct link to certain functional properties related to life history, behavioral or morphological characteristics of species that drive ecosystem functioning [2]. A better understanding of the link between species functional traits and the processes related to specific ecosystem functions (i.e., nutrient cycling, oxygen consumption, denitrification) could help increase our ability to predict the impact of fishing disturbance on the benthic ecosystem functioning.

Demersal trawls have a large footprint on the seabed [3,4]. They move over large areas, with different parts of the gears physically impacting the sediment, including the trawling wires, doors, ground rope and net [5]. The actual impact on the seabed is therefore complex, partially turning over the sediment, partially reducing spatial heterogeneity and partially increasing it on different scales [6,7,8]. At the biological level, the trawl net removes animals from bottom waters, the sediment surface and shallow sub-surface. Impact studies have shown that the abundance of macrofauna and megafauna (infaunal and epifaunal) is generally reduced with corresponding changes in the community and trophic structure [6,9,10,11,12]. Smaller body-sized fauna, however, with fast life cycles may be more resistant to trawling [8,13,14].

Sediment resuspension and sediment-water nutrient exchanges may also be strongly influenced by the mechanical effects of trawling [15,16,17,18,19,20]. The effects of trawling on biogeochemistry depend on sediment characteristics, with stronger impacts occurring on muddy sediments compared to sand [17,21]. In addition, bioturbating organisms are particularly important as agents for irrigation and the movement of oxygen into the sediment [22,23]. With trawling impacting larger species in the sediment, continually impacted areas have a lower level of bioturbation and consequently different levels of sedimentary fluxes [8,12,13,18]. Trawling was also found to cause changes in oxygen regime, influencing the nitrogen cycle, as oxygen regulates both nitrification and denitrification [15,24].

To date, there are only few studies that have investigated the impact of trawling on the relationship between specific functional traits and biogeochemical processes [17,18,21,25,26,27], primarily in Northern waters (North Sea and Baltic Sea). In the upper few millimeters of the marine sediment, the oxidation of organic carbon controls the fluxes of oxygen and nutrients across the sediment-water interface, ultimately impacting primary productivity in the water column [28]. In the highly oligotrophic Eastern Mediterranean, where regeneration of nutrients from the seabed is likely to be extremely important for pelagic productivity and resultant increases in productivity may be at some distance from regeneration sites [29,30], it is essential to reveal the role of specific traits in biogeochemical processes that take place at the sediment-water interface.

Previous work in the study area has shown that macrofauna are impacted by trawling [6,31]. As a result, it is likely that the changes in the benthic community due to trawling will also have an impact on the community functional composition that regulate the biogeochemical processes in the sediment-water interface and this impact could be chronic, particularly in areas of the seabed that are regularly trawled. The fishery in Heraklion Bay off Crete is regulated through seasonal closures during the summer months when biological activity is likely to be highest. However, it is uncertain whether this closed season is sufficient to restore biological activity at the sediment-water interface. Taking these into account, we carried out a study to test the null hypothesis that trawl fishing affects the sediment biogeochemistry linkage to specific species functional traits. This hypothesis was tested by determining oxygen flux rates, chlorophyl and organic matter concentrations in bottom water and sediment, as well as species composition and functional traits in sediment cores collected inside and outside of trawled areas during both summer (closed season) and winter (open season).

## 2. Materials and Methods

### 2.1. Study Area

**Figure 1:**
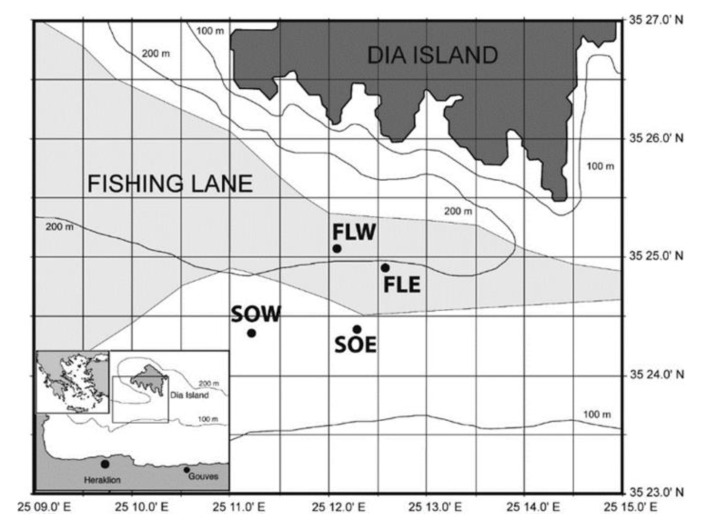
Study area showing trawling lane and control sites in Heraklion Bay, Crete (FLE, fishing lane east; FLW, fishing lane west, SOE, control area east; SOW, control area west).

The study was carried out in and adjacent to one of the main commercial trawling lanes in Heraklion Bay (Island of Crete, southern Aegean, Figure 1). The area had been identified previously and work had been carried out there for some time with respect to trawling impacts on sediment characteristics and macrofaunal community structure [6,32]. The trawling lane follows the 200 m contour and narrows with the contours behind Dia Island. This natural constriction allowed for easy identification of the trawling lane and adjacent non-trawled control areas. The surface sediments of all the sampling areas were generally similar in terms of percentage composition. Sediments were predominantly silt (85–90%) with smaller fractions of clay and sand (mostly made up of shell fragments of *Turritellinella tricarinata*, *Aporrhais serresiana* and *Nucula* sp.). At the start of each of the sampling periods, 2 days were spent surveying with side scan sonar to verify the limits of the fishing lane (areas covered with trawl door marks). Four areas were identified for bottom sampling, two control areas to the south of the trawling lane (SOE, South Out East, 190 m depth; SOW, South Out West, 215 m depth) and two areas in the trawling lane (FLE, Fishing Lane East, 185 m depth; FLW, Fishing Lane West, 230 m depth) (Figure 1). Trawling takes place between the beginning of September and the end of May followed by an annual 4 month closed period (June–September). Based on observed trawling activity and knowledge of the gear, it was estimated that the intensity of trawling in the study area was 200% (total coverage 2 times per year). It should be noted that during all the sampling there were no trawlers in the vicinity.

### 2.2. Field Data Collection

Field work was carried out at the sampling sites in Heraklion Bay during July 2002 (summer) and March 2003 (winter). From each of the four sampling areas at Dia Island, 6 individual multicore drops were made (Bowers & Connely, Oban, UK, core diameter 10 cm, core length 40 cm). From each drop, two cores were selected on the basis of good penetration, undisturbed sediment fabric and clear water over the surface. One of these cores was stored in a cool box for use in the oxygen flux determination and the other was processed for sedimentary chemical parameters. This core was sub-sampled with smaller perspex core tubes and sectioned for granulometry (4 cm diam., 0–5 cm depth), organic carbon and chlorophyll concentrations (2 cm diam., 0–2 cm). Summarizing the sampling, there was a total of 6 replicates for an east and a west trawled and untrawled area in summer and winter.

### 2.3. Physico-Chemical Analyses

Water chlorophyll and phaeopigments were determined according to the fluorometric method of Yentsch and Menzel [33] using a TURNER 112 fluorometer. The analysis of water particulate organic carbon was performed according to Parsons et al. [34] by chromic acid wet oxidation. Sedimentary organic carbon was analysed by the wet oxidation method of Walkley and Black [35]. Chlorophyllous pigments in the sediment were determined according to Strickland and Parsons [36].

### 2.4. Oxygen Flux Determination

**Figure 2:**
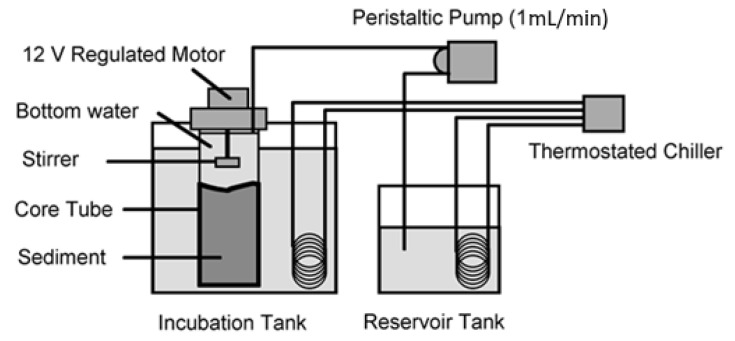
Experimental set-up for oxygen flux incubation.

The oxygen flux set-up consisted of a cold sea water recycling system (15 degrees C: stable bottom water temperature at 200 m in the area) with water flowing from a chiller unit through a cooling coil in the bottom water reservoir tank and then into the core incubation tank and back into the chiller (Figure 2). The incubated cores stood upright with the cooling water covering the major part of the core tube. Each core tube was fitted with a special head containing an electrical stirring motor, with an opening allowing overflow, sampling and insertion of an oxygen and temperature probe. The vane of each stirrer was height adjusted for each core tube and was situated near to the top of the water column such that no vortices were formed. Turning speed was approximately 1 revolution per 1.5–2 s. A 12-channel peristaltic pump (Watson-Marlow, UK) fed a constant flow (1 mL per minute) of water from the bottom water reservoir tank to the cores. When the cores were full, they overflowed into the cooling tank. Oxygen concentrations were recorded from each of the cores and the reservoir tank after 24 h of incubation. Oxygen was measured using a dissolved oxygen meter and probe (WTW Oxi-330). Oxygen flux was estimated from:

Fx = (Ci − Co) · Q/A,

where:

Fx flux of nutrient x micromol m^−2^ h^−1^)
CI concentration in the reservoir tank (μM)
Co concentration in the Core overlying water (μM)
Q flow of water through the core (l h^−1^)
A area of the core (m^2^)

Mean rates were estimated from the replicates from each sampling area.

### 2.5. Macrofaunal Community and Biological Trait Analyses

After the end of the experiment, cores were sieved through a 0.5 mm sieve for the determination of the benthic macroinvertebrate community. The fauna was sorted for species identification, enumeration and biomass measurement. A biological trait analysis (BTA) was conducted on the macrofaunal communities to determine their bioturbation attributes [37]. The biological response and effects traits considered in this study (7 traits, 27 modalities) describe the life history, morphological and behavioral characteristics of the benthic community [38] that may influence oxygen sediment-water exchanges and organic matter degradation (Table 1). Individual taxa were coded for the modalities of each trait using a fuzzy-coding procedure, which allows assessment of the affinity of a taxon to multiple categories. The trait scores were standardized for each species by re-coding the scores as percentage frequencies (Supplementary material Table S1). The species were classified into different functional categories based on information from a variety of literature sources [22,39,40,41] and databases (www.marlin.ac.uk/biotic; www.polytraits.lifewatchgreece.eu; accessed on 28 May 2021).

### 2.6. Data Analyses

To detect significant differences in environmental variables and oxygen fluxes between the samples, univariate statistical techniques were used. Specifically, three-way mixed ANOVA with trawling and season as fixed factors and site as nested factor within trawling was conducted to determine the effects of these factors on each of the variables measured (i.e., oxygen flux, organic carbon, chlorophyll a and phaeopigments). Residual analysis was performed to test for the assumptions of the three-way ANOVA. Normality was assessed using Shapiro–Wilk’s normality test and homogeneity of variances was assessed by Levene’s test. Statistical significance was accepted at the *p* < 0.025 level for simple two-way interactions and effect of trawling at each group of the other factors.

To assess the relationship between species traits and benthic biogeochemical processes as well as the effect of trawling on this relationship, a fourth-corner analysis was performed separately for the disturbed (trawled) and the undisturbed sites and for the two seasons [24,42]. The fourth-corner analysis requires three different data tables: the R table, with measurements of the environmental variables (i.e., oxygen, silt and clay content, chlorophyll a, and organic carbon); the L table, constituted by the biomass of each species in each sample; and the Q table, composed of fuzzy-coded trait data for each species. Prior to fourth-corner analysis, a standardization was applied to environmental data and variables were checked for collinearity. The Hellinger transformation was also applied to species community data. The fourth-corner method evaluates the significance of bivariate associations (i.e., one single trait and one single environmental variable at a time) [43]. For the later analysis, 49,999 permutations were used in all randomization procedures and the false discovery rate method (FDR) was selected to adjust *p*-values for multiple testing. The fourth-corner method was carried out with the ade4 package in the R program (version 4.0.2) [43,44].

## 3. Results

### 3.1. Physico-Chemical Analyses

**Figure 3:**
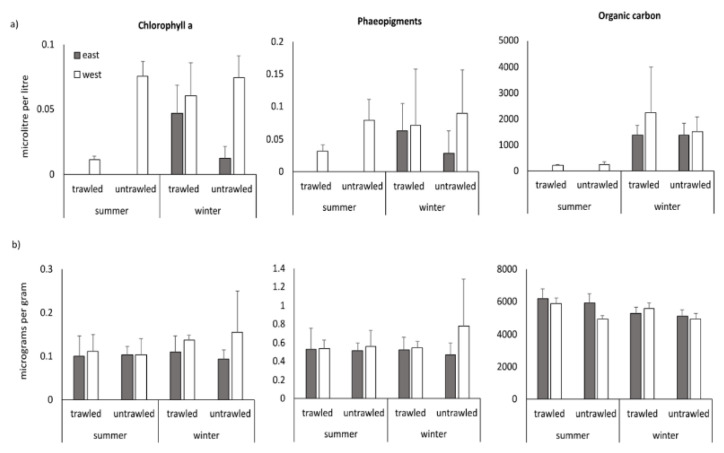
(**a**) Bottom water and (**b**) sedimentary chlorophyll a, phaeopigments and organic carbon from the Heraklion Bay trawled and untrawled sampling sites with mean and standard deviation in summer and winter. Grey bars indicate the eastern sites and white bars the western sites.

Figure 3 shows the bottom water and sedimentary parameters (no bottom water data for the easterly sampling sites during summer sampling for technical reasons). For chlorophyll a (Chl a), there was a statistically significant two-way interaction between season and trawling (Table 2). A significant effect of trawling was recorded for the summer sampling (F = 57.1, *p* = 0.000, lower values in the trawled area) but not for winter (F = 2.29, *p* = 0.138). No significant changes were recorded for the bottom water phaeopigments (Table 2). Particulate organic carbon in the bottom water varied significantly only between seasons (F = 16.51, *p* = 0.003, higher in winter).

Sedimentary Chl values were around 0.1 microg g-1 sediment. There were no significant differences between any of the factors tested (Table 2). Sedimentary phaeopigment values ranged 0.75–1.25 microg g-1 sediment, with the exception of a peak mean value in the westerly fishing lane in winter. Trends were similar to that of Chl a and there were no significant differences between any of the factors tested. A two-way interaction between site and trawling was recorded for sedimentary organic carbon (Table 2). The simple main effect of trawling was significant for the westerly sites (F = 25.6, *p* = 0.000) but not for the easterly sites (F = 1.72, *p* = 0.197). Nevertheless, the same trend (higher organic carbon in the trawled area)—even if not statistically significant—was also found in the easterly sites (Figure 3).

**Figure 4:**
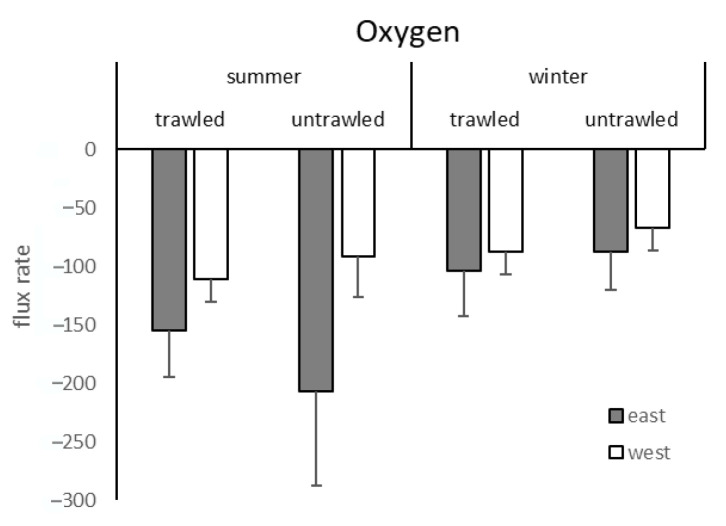
Oxygen flux rates (micromole per square metre per hour) from the trawled and untrawled sampling sites with mean and standard deviation in summer and winter. Grey bars indicate the eastern sites and white bars the western sites.

Oxygen influxes were recorded in every case (Figure 4). Trawling did not significantly affect oxygen flux rates (Table 3). Significant differences were recorded for the factors site and season (Table 2).

### 3.2. Link between Species Traits and Biogeochemical Processes

**Figure 5:**
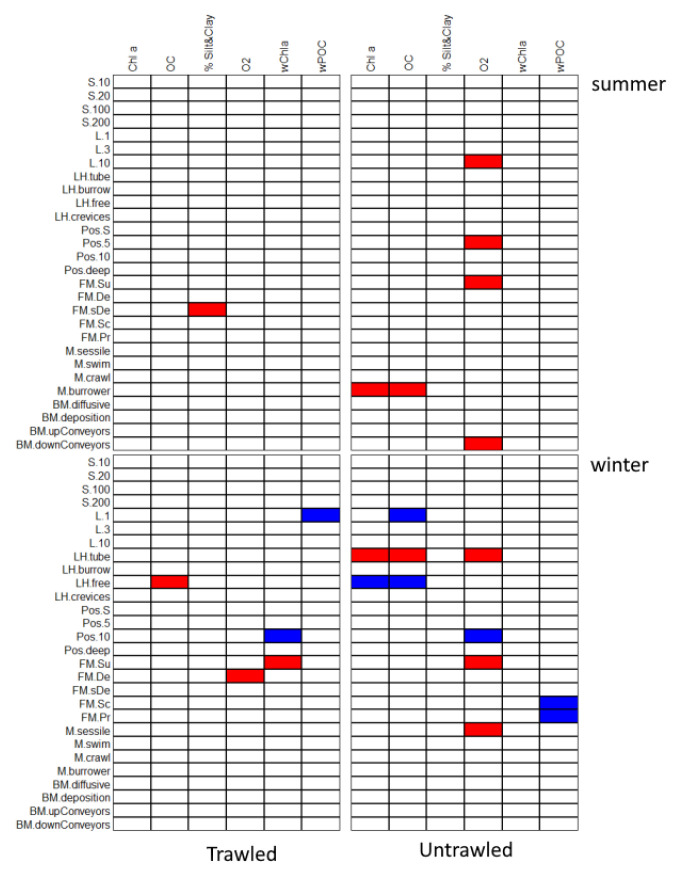
Representation of significant (*p* < 0.05) associations identified by the fourth-corner method. The blue shades indicate significant positive associations and red shades significant negative associations between traits and biogeochemical variables. Variables with no significant associations are shown in white. *p* values were adjusted for multiple comparisons using the FDR procedure. Codes for traits are explained in Table 1. Chla: chlorophyll a, OC: organic carbon, %Silt&Clay: silt and clay percentage, O_2_: oxygen, wChla: water column chlorophyll, wPOC: particulate organic carbon.

A total of 40 species were found in the samples after the end of the experiment. Their functional traits and abundances are shown in Supplementary material (Tables S1 and S2). The most abundant group was Polychaeta, followed by Bivalvia (mainly *Abra alba*), Sipuncula and Crustacea. Crustacea were not found in the cores from the trawled site nor during the trawling season (winter). The relationships between species functional traits and biogeochemical variables in different sites are summarized in Figure 5.

The significant associations (negative and positive) of species traits and biogeochemical variables describing either the bottom water or the sediment, were different in trawled and untrawled sites. More associations were recorded in the untrawled sites. The traits that are linked to biogeochemical processes in trawled sites versus control sites in both seasons are summarized in Table 3.

In the untrawled sites, biogeochemical processes are influenced by the more complex functional traits community consisting of free-living scavengers and predators, by sessile suspension feeders and also by burrowing species with high longevity that are oriented with their heads towards the sediment surface. These species transport sediment from the surface to deeper layers as they feed. In contrast, in the trawled sites biogeochemical processes were only related to short-lived species with high mobility either deposit feeders or suspension feeders.

## 4. Discussion

This study explored how trawling affects the linkage between functional traits of benthic macrofaunal species and specific biogeochemical processes such as oxygen consumption and organic matter degradation. In general, significant differences were detected only for bottom water Chl a and sediment OC concentrations between the trawled and control areas indicating that there was an impact of bottom fishing on benthic biogeochemistry. Nevertheless, oxygen consumption was not affected. In addition, the traits that are linked to specific ecosystem processes were different between trawled and undisturbed sites, highlighting the importance of unique functional traits in preserving ecosystem functioning.

The sediment and the adjacent bottom water environmental parameters could be directly affected by trawling in two ways, either removal by resuspension or constant exposure at the sediment surface by uncovering deeper sedimentary-locked carbon with continuous trawling, but also indirectly through species loss which can cause changes in ecosystem functioning [17,18,25,45,46]. As a result, differences in bottom water Chl a concentration and sedimentary organic carbon at the different sites can be explained by the aforementioned processes. Pusceddu et al. [47] in the northwestern Aegean, found that sedimentary organic carbon concentrations displayed a significant increase immediately after the initiation of the trawling season, probably from trawling-induced uplift from deeper sediment layers, but no significant short-term changes in sedimentary pigment levels. In accordance, Sciberras et al. [17] have linked bottom trawling to increased sediment Chl a and organic carbon and attributed this enhancement to a considerable reduction in bacterial biomass due to sediment resuspension that leads to a slow-down in the remineralization of the labile portion of organic matter within the sediment rather than to a loss in macrofaunal community bioturbation potential. Despite the findings of Sciberras et al. [17], macrofaunal species play also an important role in controlling the levels of OC within the sediment via bioturbation [48]. Thus, the difference in OC between the trawled and untrawled areas may be related to differences in macrofauna functional traits. Specifically, a decline in community complexity and bioturbation capacity can lead to a decrease in sediment oxygenation and carbon cycling and result in higher sedimentary OC concentrations [48]. On the other hand, oxygen consumption remains unaffected by trawling in our experiment which is in accordance with other studies suggesting that oxygen consumption is either unaffected [15,17] or decreased [18,19,21] by trawling due to the removal of the reactive surface sediment and the consequent reduction in carbon mineralization.

Besides the changes in environmental parameters, trawl fishing causes changes in species composition either through the direct removal of animals or by decreasing the settlement succession of species with pelagic larvae [49] from the control areas in the trawled areas and vice versa. This may result in alterations both in the functional effects and response traits of a community, that in turn may have broad implications for the overall ecosystem functioning [50]. In our study, trawling affected the links between species traits and biogeochemistry. Specifically, the untrawled sites presented more variable associations between specific traits and biogeochemical processes than the trawled sites. This indicated that in the undisturbed area, biogeochemistry was mainly controlled by macrofaunal community. In contrast, trawled sites appeared to have less associations between macrofaunal traits and biogeochemical variables. This could be explained either by lower functional redundancy within the disturbed sites due to fewer species overall or by the stimulation of microbial activity against the macrofaunal-induced metabolism in the disturbed sites [18,24,51]. It is also worth noting that more associations between species functional traits and biochemical processes were recorded in winter than in summer. It was anticipated that higher temperatures in summer would have favored macrofaunal activity [52] and as a result, an increased number of significant associations between functional traits and specific biogeochemical processes. Nevertheless, there is not always a straightforward relation between single species and biogeochemical cycling processes but rather complex interactions of species competition for space and food and habitat characteristics since macrofaunal activities have different effects across different environments [53].

In the undisturbed sites, many traits were found to be related to benthic biogeochemistry indicating a balanced and diverse community. Bioturbation, burrowing activities and feeding mode are some of the traits influencing sediment geochemistry. Specifically, bioturbation and burrowing affect nutrient fluxes across the sediment water interface, organic carbon concentrations, chlorophyll burial and decomposition, oxygen consumption and mediation of nitrogen [22,24,54,55,56,57,58]. In addition, sessile species that live in tubes influence the stability and accumulation rates of sediment, which are key drivers of organic carbon burial and storage [28,59]. Burrowing fauna can increase the stability and accumulation rate of sediment if there is an increase in biogenic materials such as tubes or mucus production [60].

In contrast to the undisturbed sites, long-lived bioturbators, sessile and burrowing fauna that are particularly vulnerable to damage from mobile demersal fishing, in the trawled sites showed reductions due to the resuspension and the following deposition of the surface sediments caused by trawling [8,18,61]. Subsequently, small opportunistic deposit feeders with low bioturbation ability may take advantage of the aforementioned reduction and appeared to be linked with the biogeochemical parameters in the trawled sites. Thus, under trawling disturbance, the shift towards opportunistic lifestyles can decouple the macrofauna-sediment relations that facilitate nutrient cycling and lead to a microbially driven metabolism [17,51,60].

## 5. Conclusions

Trawling had an impact on the links between macrofaunal functional traits and biogeochemical processes. Nevertheless, this impact was not adequately reflected in all the biogeochemical variables studied. As a result, in the trawled sites, either there was an uncoupling of species-sediment relations towards a microbially induced metabolism or the benthic community, and specifically ephemeral deposit feeders, took advantage of the organic matter availability due to sediment resuspension and preserved benthic biogeochemistry. The differences recorded in the associations of species functional traits and biogeochemical processes in both trawled and untrawled sites underlined the importance of unique functional traits on ecosystem functioning. In addition, in such an experimental design, quantitative effects of trawling are likely to be underestimated due to reduced suitable settling area for species also within the undisturbed area due to its spatial vicinity to the trawled sites.

Finding the links and relating species functional traits to important specific ecosystem processes will help scientists and policymakers to better predict and communicate the impact of fishing disturbance on benthic ecosystem functioning and set appropriate thresholds for adverse effects. Exceeding these thresholds should trigger management response actions. To this end, successful management measures could include confining the trawling footprint within historically trawled areas and/or allocating a longer closed period for trawling to restore the area [62].

## Figures and Tables

**Table 1:** Description of traits and trait modalities used in the biological trait analysis.

	Trait	Modalities	Code	Trait Definition
Effect traits	Maximum body size (length)	<10	S.10	<10 mm
		11–20	S.20	11–20 mm
		21–100	S.100	21–100 mm
	Longevity (max)	<1	L.1	<1 year
		1–3	L.3	1–3 years
		3–10	L.10	3–10 years
	Feeding mode	Suspension & filter	FM.suspension	obtains food from water
		Surface deposit	FM.sdeposit	including grazers
		Sub-surface deposit	FM.subsdeposit	sub-surface deposit
		Scavenger/opportunist	FM.scavenger	feeds upon dead animals
		Surface predator	FM.predator	actively predates upon animals
	Bioturbation mode	Diffusive mixers	BM.diffusive	vertical and/or horizontal movement of sediment or particles, organisms with activities that result in a constant and random local sediment biomixing over short distances
		Surface depositors	BM.deposition	deposition of particles at the sediment surface, species whose activities are restricted to <1–2 cm of the sediment
		Upward conveyors	BM.upConveyors	upwards movement of particles resulting from biological activity, head down feeders that actively transport sediment to the sediment surface
		Downward conveyors	BM.downConveyors	downwards movement of particles resulting from biological activity, head up feeders that actively transport sediment from the sediment surface
Response traits	Living habit	Tube-dwelling	LH.tube	builds a tube
		Burrow-dwelling	LH.burrow	builds a burrow, includes mucus-lined burrows
		Free-living	LH.free	freely moves around sediment/water
		Inhabits crevices	LH.crevices	Inhabits crevices/holes/under stones
	Sediment position	Surface	Pos.S	surface dwellers
		Infauna: 0–5 cm	Pos.5	shallow-dwellers
		Infauna: 6–10 cm	Pos.10	buried deeper
		Infauna: >10 cm	Pos.deep	deep-dwelling
	Mobility	Sessile	M.sessile	immobile, fixed in a place, stalked or not
		Swim	M.swim	includes those which may stop swimming temporarily
		Crawl/creep/climb	M.crawl	those which move above bed slowly
		Burrower	M.burrower	Infers relatively low mobility

Traits are categorized in effect and response traits. Response traits refer to functions related to the ability of the organism to survive and effect traits refer to how organisms influence the environment.

**Table 2:** Three-way ANOVA results for the comparisons of the bottom water and sedimentary environmental variables as well as oxygen flux for the factor trawling, season and site. Significant differences at *p* < 0.05 indicated with “*”, at *p* < 0.01 indicated with “**” and non-significant indicated with “ns”.

		Bottom Water						Sediment						Flux	
		Chlorophyll a		Phaeopigments		Organic Carbon		Chlorophyll a		Phaeopigments		Organic Carbon		Oxygen	
Source of Variation	DF	F	*p*	F	*p*	F	*p*	F	*p*	F	*p*	F	*p*	F	*p*
trawling	1	22.55	**	1.40	ns	0.75	ns	0.02	ns	0.31	ns	116.96	**	0.03	ns
season	1	17.93	**	1.38	ns	16.51	**	1.30	ns	0.23	ns	16.30	**	33.84	**
site	1	-	-	-	-			2.71	ns	2.04	ns	9.55	*	16.53	**
season × trawling	1	19.80	**	0.49	ns	0.92	ns	0.02	ns	0.47	ns	0.51	ns	3.55	ns
trawling × site	1	-	-	-	-	-	-	0.29	ns	1.68	ns	8.46	*	2.23	ns
season × site	1	.	-	-	-	-	-	3.62	ns	1.57	ns	8.85	*	2.23	ns
trawling × season × site	1	.	-	-	-	-	-	0.33	ns	0.46	ns	0.18	ns	0.53	ns

**Table 3:** Summary of the major functional groups related (positively: + and negatively: −) to specific biogeochemical processes in different trawling intensities and seasons. OC: organic carbon, POC: particulate organic carbon.

Season	Trawling		Description	Sediment Chl *a*	OC	Silt and Clay	Oxygen	Water Chl *a*	POC
summer	untrawled	Group 1	long-lived infauna, burrowers and suspension feeding species	−	−		−		
winter		Group 2	sessile species that live in tubes and suspension feeders	−	−		−		
		Group 3	free living infauna that moves in the sediment, scavengers and predators	+	+		+		+
summer	trawled	Group 4	deposit feeding species			−			
winter		Group 5	free living infauna that moves in the sediment, deposit feeders		−		−	+	+
		Group 6	Suspension feeding species					−	

## Data Availability

Data on the benthic community can be found in the Supplementary Materials.

## Supplementary Materials

The following supporting information can be downloaded at: https://www.mdpi.com/article/10.3390/biology11101378/s1, Table S1: Species trait scores, Table S2: Species abundance per sampling core, Table S3: ANOVA results.
